# Targeted Capture of Homoeologous Coding and Noncoding Sequence in Polyploid Cotton

**DOI:** 10.1534/g3.112.003392

**Published:** 2012-08-01

**Authors:** Armel Salmon, Joshua A. Udall, Jeffrey A. Jeddeloh, Jonathan Wendel

**Affiliations:** * Department of Ecology, Evolution, and Organismal Biology, Iowa State University, Ames, Iowa 50011; † Department of Plant and Wildlife Sciences, Brigham Young University, Provo, Utah 84602, and; ‡ Roche NimbleGen Inc., Madison, Wisconsin 53719

**Keywords:** *Gossypium*, allopolyploidy, homoeologs, sequence capture, next-generation sequencing

## Abstract

Targeted sequence capture is a promising technology in many areas in biology. These methods enable efficient and relatively inexpensive sequencing of hundreds to thousands of genes or genomic regions from many more individuals than is practical using whole-genome sequencing approaches. Here, we demonstrate the feasibility of target enrichment using sequence capture in polyploid cotton. To capture and sequence both members of each gene pair (homeologs) of wild and domesticated *Gossypium hirsutum*, we created custom hybridization probes to target 1000 genes (500 pairs of homeologs) using information from the cotton transcriptome. Two widely divergent samples of *G. hirsutum* were hybridized to four custom NimbleGen capture arrays containing probes for targeted genes. We show that the two coresident homeologs in the allopolyploid nucleus were efficiently captured with high coverage. The capture efficiency was similar between the two accessions and independent of whether the samples were multiplexed. A significant amount of flanking, nontargeted sequence (untranslated regions and introns) was also captured and sequenced along with the targeted exons. Intraindividual heterozygosity is low in both wild and cultivated Upland cotton, as expected from the high level of inbreeding in natural *G. hirsutum* and bottlenecks accompanying domestication. In addition, levels of heterozygosity appeared asymmetrical with respect to genome (A_T_ or D_T_) in cultivated cotton. The approach used here is general, scalable, and may be adapted for many different research inquiries involving polyploid plant genomes.

Recent and dramatic advances in sequencing technologies have been revolutionizing many areas in biology. For some types of evolutionary and population-level analyses, whole-genome sequencing is either unnecessary or unfeasible, in which case recently developed targeted sequence enrichment methods have become quite attractive. These methods enable efficient and relatively inexpensive sequencing of hundreds to thousands of genes or genomic regions from many more individuals than is reasonable using whole-genome sequencing approaches. Current target enrichment strategies include DNA capture using microarrays [*e.g.*, NimbleGen (Albert *et al.* 2007) or Agilent (Porreca *et al.* 2007)], DNA capture on beads (*e.g.*, Agilent SureSelect, NimbleGen SeqCap EZ, Mycroarray Inc), and amplicon-based methods (*e.g.*, Fluidigm AccessArray and Molecular Inversion Probes). The advantages and disadvantages of these methodologies have recently been reviewed (Cronn *et al.* 2012; Grover *et al.* 2012; Mamanova *et al.* 2010; Mertes *et al.* 2011), although applications in plants remain relatively few in number (Fu *et al.* 2010; Haun *et al.* 2011; Saintenac *et al.* 2011). Because plant genomes are rife with duplications large and small, it is important to develop targeted sequencing approaches that facilitate the assignment of multiple homologous sequences into the appropriate categories of homeology, orthology, and paralogy. In addition, it would be useful to design experiments that enable the capture of flanking sequence and introns based on more limited information such as that obtained from expressed sequence tag (EST) libraries or assemblies, especially for lineages in which genomic resources (*i.e.*, reference sequences) are lacking. In this brief report, we demonstrate these two dimensions of targeted sequence capture for the allopolyploid *Gossypium hirsutum*, which is the most economically important of the four domesticated cotton species.

Allopolyploid cottons comprise a clade of five species containing two coresident genomes, designated A_T_ and D_T_, where the ‘T’ subscript indicates the diploid progenitor-of-origin, *i.e.*, A or D. Diploid A-genome (*Gossypium arboreum*) and D-genome (*Gossypium raimondii*) species are closely related to their counterparts in the allopolyploid nucleus, as demonstrated by both DNA sequencing and comparative cytogenetic techniques (Endrizzi *et al.* 1985; Brubaker and Wendel 1994; Senchina *et al.* 2003; Flagel *et al.* 2012). Because the average genic exons differ by less than 1% in sequence in A_T_ and D_T_ homeologous genes, we hypothesized that it would be possible to simultaneously target the sequence of both genomes by sequence capture. With successful simultaneous captures, we could assess: (1) whether sequence capture probes designed using EST contigs from an unsequenced genome would sufficiently enrich target loci; (2) evaluate whether unique identifiers could be multiplexed to ‘barcode’ 454 sequencing reads; (3) determine whether multiple samples of a polyploid could be effectively pooled before hybridization thereby allowing a single sequencing reaction; and (4) assess whether capture and sequencing efficiencies differed for two diverse accessions (the wild *G. hirsutum* accession TX2094, and the cultivar Maxxa Acala) of the same polyploid species.

Here, we demonstrate the feasibility of target enrichment using sequence capture in polyploid cotton. We created custom hybridization probes to target 500 pairs (homeologs) of selected genes based on information from the cotton transcriptome. Collectively, this set of loci represents approximately 550 kb of haploid transcript space. Two widely divergent samples of *G. hirsutum* were hybridized to four capture arrays containing capture probes. We show that the two coresident homeologs in the allopolyploid nucleus were equally captured with high coverage, that capture efficiency was similar between the two accessions and independent of whether the samples were mixed or jointly hybridized, and that a significant amount of flanking, nontargeted sequence (untranslated regions [UTRs] and introns) also was captured and sequenced along with the targeted exons. These results offer a general approach that could be adapted for all complex plant genomes.

## Materials and Methods

### Genomic MID-tagged DNA libraries

Two DNA samples were each extracted from young leaves of two accessions (TX2094 and Maxxa Acala, afterward simply referred to as Maxxa). The Roche GS FLX Titanium DNA Library Preparation kit was used to prepare four whole-genome shotgun fragment libraries for 454 sequencing. Samples were nebulized, and the fragments were end-repaired. Multiplex Identifier 1 (MID1) and MID2 were separately ligated (along with sequencing adapters) to the fragments of one library of Maxxa and one library of TX2094, respectively. The remaining two libraries (Maxxa and TX2094) did not have MID-encoded adapters attached to the fragments. The ligated libraries were then size-selected using the gel-cut technique (fragment size between 500 and 650 bp), and the libraries were then amplified (11 cycles) using the Ti-A and Ti-B primers (Table 1) to obtain sufficient DNA quantity for hybridization (100 ng). The quality and the concentration of the prepared libraries were checked on a Bioanalyzer (Agilent Bioanalyser DNA 7500 Pico Chip) and a Nanodrop.

**Table 1:** Roche NimbleGen MID-adaptors, hybridization-enhancing oligomer sequences, and qPCR primers used for the postcapture enrichment estimation of the DNA libraries

Oligonucleotide	Oligonucleotide Sequence
	MID-tags
MID1	ACGAGTGCGT
MID2	ACGCTCGACA
	LM-PCR primers
LM-PCR 454 Ti-A	CCATCTCATCCCTGCGTGTC
LM-PCR 454 Ti-B	CCTATCCCCTGTGTGCCTTG
	Hybridization-enhancing oligonucleotides
Ti-A−enhancing	CCATCTCATCCCTGCGTGTCCCGACTCAG/3ddc/*^a^*
MID1- Ti-A−enhancing	CCATCTCATCCCTGCGTGTCTCCGACTCAGACGAGTGCGT/3ddc/*^a^*
MID2- Ti-A−enhancing	CCATCTCATCCCTGCGTGTCTCCGACTCAGACGCTCGACA/3ddc/*^a^*
Ti-B−enhancing	CCTATCCCCTGTGTGCCTTGGCAGTCTCAG/3ddc/*^a^*
	qPCR primers (forward/reverse primers)
Target 1 (Cotton16_00329_02)	CCAGCAATCCCAAACAAGAT/CTTCCCTTGGGACATAGCAA
Target 2 (Gh_MYB109)	GGAAGTCATTGATCATCGGG/TGGTCAATCATGGCAAGTGT
Target 3 (Gh_CAPRICE)	GTGGGCTTTAATTGCTGGAA/TAATTGCCATCCAGTCCCTC
Target 4 (Cotton16_00219_02)	GTAGTGTACGCGCCAGTTGA/GTTTCCGCTAACATGTCCGT
Untargeted (Gh_ndhF)	AGCCCTTGCTCAAAACGATA/TCGATCCGGATGCTAAAAAC

MID, Multiplex Identifier; qPCR, quantitative polymerase chain reaction; LM, ligation-mediated.

a /3ddc/: 3′ dideoxy-C modification.

### NimbleGen capture array design

Five hundred pairs of genes were targeted for enrichment based on previous studies of gene expression and from published literature of cotton. The results from gene expression experiments of developing cotton fiber and salt-stress were cross-tabulated so that both highly expressed and differentially expressed genes could be selected (Chaudhary *et al.* 2008; Hovav *et al.* 2008; Rapp *et al.* 2010; J. A. Udall, personal communication). Additional genes were chosen based on studies of cotton fiber development and of trichome development in *Arabidopsis*. We also included members of several transcription factor gene families (MYB, TCP, and WRKY, based on Blast annotations). The list of targeted genes (supporting information, Table S1) is available at http://cottonevolution.info. The length of the targets designed from EST assemblies ranged from 335 to 3377 bp with a mean length of 1085 bp (SD 479.8) and a total length of approximately 550 kb. Hybridization probe-sets for the selected targets were designed from consensus sequences calculated from A-genome, D-genome, and AD-genome ESTs sequenced from various cDNA libraries described in Udall *et al.* (2006) and by Roche NimbleGen using proprietary software algorithms with generalized parameters for probe sequence, hybridization temperature, and length (considering that the cotton genome sequence was not available). The probes designed for each of the 500 pairs of targets overlapped 465,034 bp, or 85.6% of the targeted sequences, and were synthesized on HD2 (HX12) arrays, each slide consisted of 12 subidentical subarrays each with 135,000 probes designed against the 550-Kbp transcript space. The individual subarrays were not used *per se* in this experiment; rather, they provided a perspective on enrichment efficiency with this platform in mind for future experiments. While this paper was being written, a draft genome sequence of *G. raimondii* (D_5_) was released (DOE Joint Genome Institute: Cotton D V1.0, available at http://www.phytozome.net/cotton.php). We inferred the copy number of the targeted genes by searching for the sequences used for probe design within the genome sequence (Table S2). A total of 411 of the 500 targeted genes appeared to be single-copy.

### Microarray hybridization and elution of captured libraries

**Figure 1:**
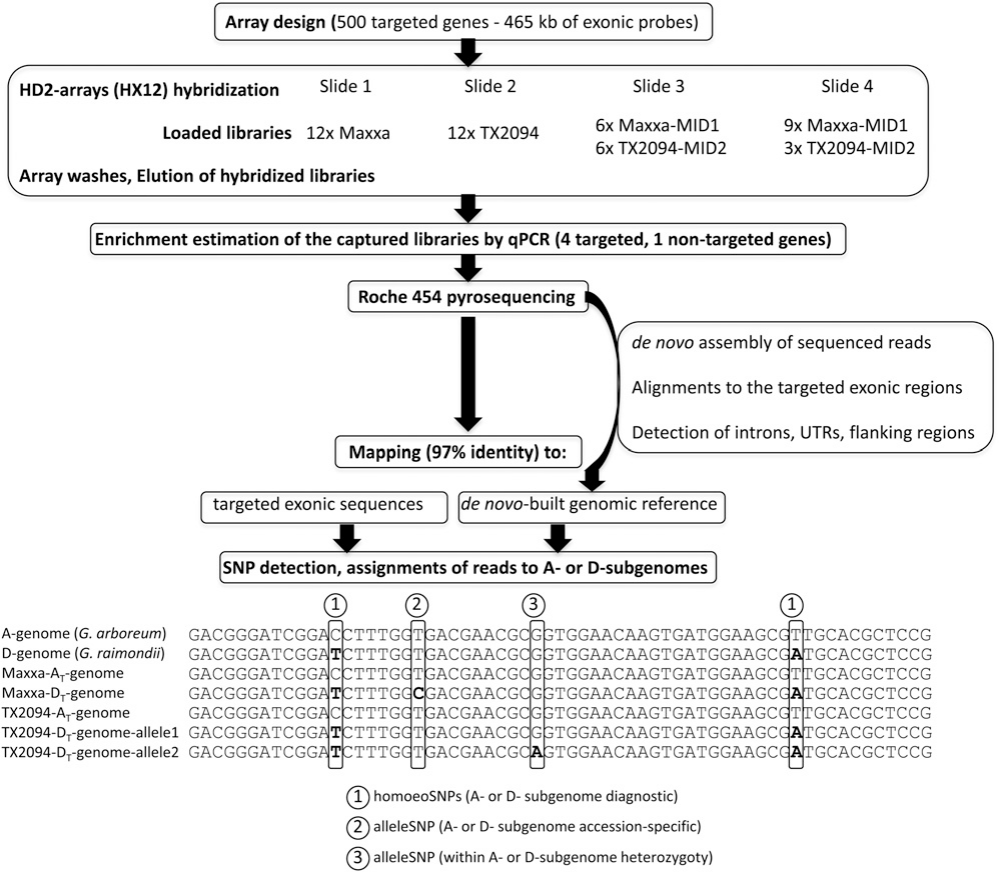
Overall workflow for targeted sequencing of 500 genes in cotton.

A total of four HX12 slides (NimbleGen) were used, each consisting of 12 subarrays containing 135,000 probes designed from the 500 targeted genes. The Maxxa and TX2094 libraries (without MIDs) were separately hybridized to one slide each (*i.e.*, each of the 12 wells received the same library; Figure 1). A second pair of arrays was subjected to precapture library multiplexing and different mixture ratios of the Maxxa and TX2094 libraries to estimate the relative enrichment and capture efficiency of multiplexed MID-tagged libraries. One slide was loaded with a 50:50 ratio of the two MID-labeled libraries (of the subarrays, six wells loaded with one library and six wells loaded with the other) and the other with a 75:25 mix (Figure 1).

The samples were prepared as following for microarray hybridization: 100 μg of DNA library, 20 µL of Plant Capture Enhancer solution (Roche NimbleGen), and 20 nmoles of Hybridization Enhancing A and B oligonucleotides were dried at 60° in a SpeedVac. Samples were resuspended in 4.8 µL of water and heated at 70° to fully solubilize the libraries. To each sample, 8µL of 2X SC Hybridization Buffer (Roche NimbleGen) and 3.2 µL of SC Hybridization Component A (Roche NimbleGen) were added. DNAs were denatured at 95° for 10 min and placed at 42° until ready for hybridization. The slides were assembled according to NimbleGen instructions. Six microliters of each sample was loaded for each subarray and the hybridization performed on a NimbleGen Hybridization System 4 (mix mode B) at 42° for 72 hr. After hybridization, the slides were washed and eluted as previously described (Fu *et al.* 2010). The eluted captured libraries were then titer-amplified using the Ti-A and Ti-B primers (Table 1). Titer-amplification sampled a single, preliminary 36 µL of polymerase chain reaction (PCR) for each elute for 8, 10, 12, 14, and 16 cycles. The library amplification product was visualized on a Lonza gel, and the number of cycles for each library was individually determined. Once the number of cycles was identified, 16 PCRs (50 µL) were completed using high-fidelity *Taq* DNA polymerase (Clontech), and the products were cleaned using a QiaQuick column (QIAGEN).

### Enrichment estimation by quantitative PCR and 454 sequencing

To evaluate the enrichment of the captured libraries before sequencing, a quantitative PCR (qPCR) assay was performed for four targeted and one nontargeted genes (Table 2). Three replicates were used for each gene, for each accession, using both the precapture and postcapture libraries. The qPCRs contained 5 ng of pre- or postcapture genomic libraries, 1X of SYBR Green Master I (Roche), 0.5 µM of each primer in a final volume of 10 µL. The qPCR assays were amplified on the LightCycler 480 II (Roche) for 40 cycles followed by a melting curve analysis to quantify amount of target enrichment by sequence capture. PCR efficiencies (E) were measured for each locus, and the fold-enrichment was calculated using the difference between the mean crossing point (CP) values reported between the precapture and the postcapture matrices (∆_CP_), with: fold enrichment = E^∆CP^. The quality and the concentration of the enriched libraries were checked on a Bioanalyser DNA7500 chip (Agilent) and on a Nanodrop, and the libraries were diluted for emulsion PCR as recommended by 454 Life Science and sequenced using a single 4-region Titanium PicoTilterPlate (PTP; 1 region per capture array) on a GS-FLX Titanium 454 Sequencing System (DNA Sequence Center, Brigham Young University, Provo, UT).

**Table 2:** Postcapture enrichment estimations

Gene	Capture*^a^*	Mean CP Precapture	Mean CP Postcapture	∆-CP	Fold Enrichment
Cotton16_00329_02	1	16.90	23.82	6.92	93.93
	2	16.59	23.26	6.67	79.71
	3	16.09	23.21	7.12	107.12
	4	NA	23.57	NA	NA
MYB109	1	14.35	22.11	7.76	176.08
	2	13.99	21.79	7.80	181.24
	3	14.15	21.82	7.67	166.19
	4	13.64	21.71	8.07	216.5
CAPRICE	1	16.99	22.31	5.32	32.34
	2	17.00	21.88	4.88	24.31
	3	NA	22.31	NA	NA
	4	16.21	21.88	5.67	40.74
Cotton16_00219_02	1	14.89	22.46	7.57	189.57
	2	14.76	21.92	7.16	143.01
	3	14.69	22.61	7.92	242.18
	4	13.94	22.25	8.31	317.35
ndhF	3	19.94	13.69	6.25	0.01
(Nontargeted)	4	19.61	14.21	5.40	0.02

CP, crossing point; NA, not available.

a Capture libraries are as follows: 1 = Maxxa; 2 = TX2094; 3 = Maxxa-MID1 + TX2094-MID2 (50:50); 4 = Maxxa-MID1 + TX2094-MID2 (75:25).

### Sequence assembly

A *de novo* assembly of the captured reads was performed to discover the intronic and untranslated regions of the captured genomic regions hybridized to exonic probes (GS *De novo* Assembler Software v.2.5.3; Roche). The resulting *de novo* assembled contigs were assigned to the target exonic sequences using BLASTN (Altschul *et al.* 1990). We assumed that the best blast hit (at a minimum of 95% sequence similarity) would be the correct match between the *de novo* assembled contig and the reference gene. Spidey (http://www.ncbi.nlm.nih.gov/IEB/Research/Ostell/Spidey/index.html) was used to align genomic (*de novo* contigs) to exonic sequence (reference exonic sequence). The alignments between the best match *de novo* contigs and the reference transcripts led to the detection of large gaps corresponding to the genomic untranslated regions (*i.e.*, UTRs and introns) and flanking exonic regions. Intron/exon boundaries were verified using the ghmme3 program of genemark [*Arabidopsis* splice junctions were used as a reference (Besemer and Borodovsky 2005)].

The full set of 454 reads (both MID-labeled and nonlabeled reads from both accessions) were mapped to two reference sequences of the 500 targeted pairs of homeologs: (1) the exonic EST templates used for capture-probe design; and (2) a genomic reference rebuilt from the *de novo* assembled contigs. Different minimum overlap identity rates (90%, 95%, and 97%) were tested, which also facilitated measuring on- and off-target rates for each library and each experiment. The results presented here were obtained with a 97% mapping stringency to avoid the comparison of putative close paralogs.

### Single-nucleotide polymorphism detection

Alignments to the reference sequences were scanned (using the Ace.py program from biopython, http://biopython.org/ and custom python scripts) for two types of single-nucleotide polymorphism (SNPs; Figure 1): (1) genome-specific SNPs that differentiated the A_T_- and D_T_-genomes at homeologous positions (hereafter, homoeoSNPs); and (2) Allelic-SNPs that represented single-base differences within the homeologous copies detected (hereafter alleleSNPs). HomoeoSNP positions were determined by comparing the bases of simultaneously mapped EST orthologs from the A_2_ and D_5_ diploid species to the sequence references (available at http://cottonevolution.info). We assigned the tetraploid captured reads to either the A_T_- (A-genome within the tetraploid) or D_T_-genome by individually comparing their bases at homoeoSNP positions to the diploid bases at that position. The minority homoeoSNP was required to be present in a minimum of three reads for each tetraploid genome and in 100% of the reads of the orthologous diploid relatives. Two types of alleleSNPs were detected (Figure 1). First, alleleSNPs that distinguish between Maxxa and TX2094 were identified in each of the two coresident genomes. Second, SNPs were detected within Maxxa or TX2094 (*i.e.*, variable within Maxxa A_T_-genome, variable within the Maxxa D_T_-genome, variable within the TX2094 A_T_-genome, and variable within the TX2904 D_T_-genome). These SNPs within A_T_-genomes or D_T_-genomes indicate where the accessions were heterozygous. In each case, the minor SNP allele was required to have >5x coverage and be present in 30% of the reads. Sequencing errors detected as rare variants or within homopolymeric regions (repeats of more than 4 times the same nucleotide presenting putative false-positive SNPs) were removed from our analyses.

## Results

### Fold enrichment of targeted loci

**Figure 2:**
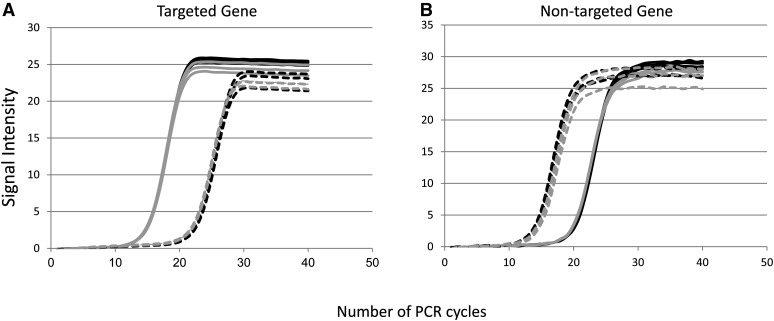
Amplification curves pre- (dashed lines) and postcaptures (solid lines) from the captures of the two accessions tested (Maxxa, in gray; TX2094 in black) for a targeted gene, *GhMYB109* (A) and a nontargeted gene, *ndhF* (B).

Successful targeted capture and sequencing results in a library of sequences that is enriched for the loci of interest. The extent to which the target region(s) are enriched is a direct measure of the efficacy of the sequence capture procedure. Typically this measurement is reflected as the proportion of total reads from the target region divided by the fraction of the genome the targeted region comprises. Because this calculation requires sequencing the samples, often an assay such as quantitative PCR is used as a first-pass surrogate measure of experimental success. In our case, the fold-enrichment estimation of the capture experiment assessed by qPCR for four targeted genes (Cotton16_00329_02, GhMYB109, GhCAPRICE, and Cotton16_00219_02) varied from 24- to 317-fold, depending on the gene tested and the capture assay (Table 2). The qPCR curves depicted (Figure 2) illustrate the effect of the capture on the enrichment of the targeted genes and the expected depletion of a nontargeted gene (*ndhF*) that is estimated up to 100× (enrichment factor of 0.01; Table 2). Complicating our analysis was the fact that the source of our capture template was exonic, meaning that the intronic space was unaccounted for our fold enrichment estimates. Because all four positive controls (enrichment loci), and the negative control (depletion locus) exhibited the proper enrichment/depletion relationships, we sequenced the libraries using 454 sequencing.

### Enrichment of 454 sequenced captured libraries and MID tag recovery

The 454-sequencing run of the captured libraries resulted in 1,017,064 reads (available at http://cottonevolution.info). MID-tagged libraries from the two multiplexing assays (in two different 1/4 PTPs) were recovered from the total sequenced reads at approximately the expected proportion: for the 50:50 MID-tagged library multiplexing assay, the MID-tagged reads represent 42.2% and 57.8% of the sequenced reads for the MID1- and MID2-tagged libraries respectively, whereas for the 75:25 MID-tagged library multiplexing assay, the MID-tagged reads represent 75.7% and 24.3% of the sequenced reads for the MID1 and MID2-tagged libraries, respectively (Table 3). Considering pipetting and sampling error, these small deviations from the expected ratios were considered acceptable.

**Table 3:** Number of reads captured and mapped at a minimum overlap identity of 90% on the targeted probes

	Number_of_Reads (Relative Percentage)	Number_of_Mapped_reads (Percentage)
Maxxa	246,719	116,530 (0.47)
TX2094	229,173	102,204 (0.44)
Maxxa-MID1 (50:50 assay)	114,413 (0.42)	69,535 (0.60)
TX-2094-MID2 (50:50 assay)	156,445 (0.58)	50,259 (0.32)
Maxxa -MID1 (75:25 assay)	204,668 (0.76)	33,557 (0.16)
TX-2094-MID2 (75:25 assay)	65,646 (0.24)	31,569 (0.48)
	1,017,064	403,654 (0.45)

**Figure 3:**
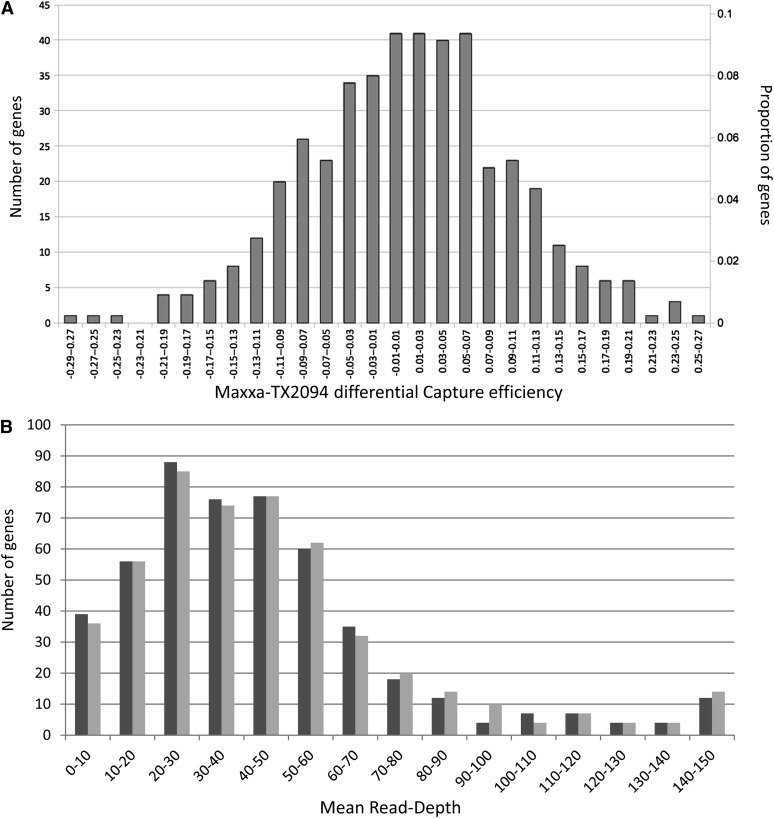
(A) Average distribution of the differential capture efficiencies between Maxxa (untagged and MID1 libraries) and TX2094 (untagged and MID2 libraries) normalized per quarter capture array. The number of genes and proportion of genes are on the y-axes, respectively. (B) Distribution of the mean coverage for Maxxa (in light gray) and TX-2094 (in dark gray) of the captured reads mapped to the 500 targeted gene pairs.

Direct mapping of the captured libraries resulted in 403,654, or 39.7%, of the total reads mapping to the targeted sequences, the proportion ranging from 16.4% to 60.8% depending on the captured library. Little capture efficiency difference was observed for the two accessions tested (Figure 3A). Greater than 98% of the targeted genes exhibited a differential capture which differed by less than ±20% between Maxxa and TX2094 (Figure 3A), illustrating the efficiency of simultaneous capture of different accessions (with or without MID-tags). We examined the distribution of mean coverage of the captured reads mapping to the 500 targeted gene pairs: more than 400 gene pairs (407 and 404 for Maxxa and TX2094, respectively) are represented by a mean read depth of more than 20X, which was our goal for identifying homeologous copies and alleles in allopolyploid cottons (Figure 3B). One targeted gene pair (Cotton16_41254_01, coding a putative tRNA) was highly overrepresented, with 152,398 reads. Inadvertent inclusion of this highly repeated sequence (its copy number in the unpublished, draft D-genome is 136) in the capture design significantly lowered the resulting sequence coverage for the other targeted genes. Because of its relatively high genomic copy-number, this sequence was removed from subsequent analyses of single-copy genes.

### Recovery of adjacent and intervening regions not targeted

**Figure 4:**
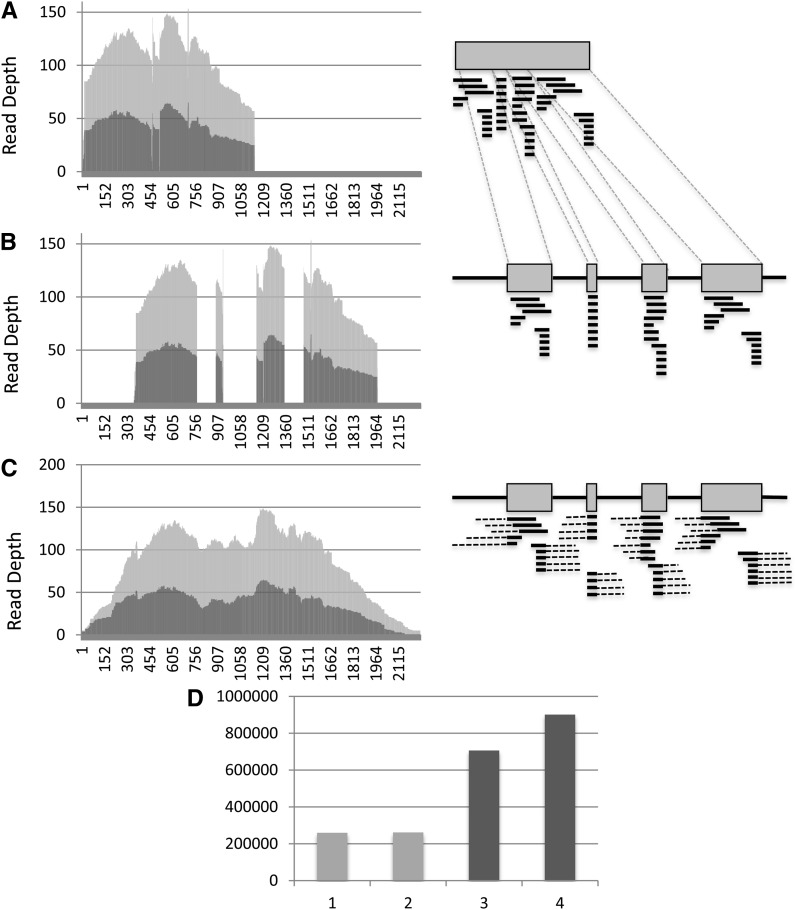
Genomic reference sequence reconstruction and its effects on mapping results for the Cotton16_31308_01 EST contig. (A−C) left: read depth per position for Maxxa (light gray) and TX2094 (dark gray); right: illustration of the mapped reads. (A) Reads mapped to the target sequences. (B) Reads mapped to different exonic parts of a gene when visualized against the reconstructed genomic reference. (C) Flanking genomic sequence detected using *de novo* assemblies mapped to the probe sequences and reads mapped to the genic space could be used to reconstruct the genomic sequence. (D) 1, number of mapped reads based on the targeted sequences; 2, number of mapped reads based on the recovered genomic reference; 3, number of mapped bases (divided by 100) based on the targeted sequences; and 4, number of mapped bases (divided by 100) based on the recovered genomic reference.

**Figure 5:**
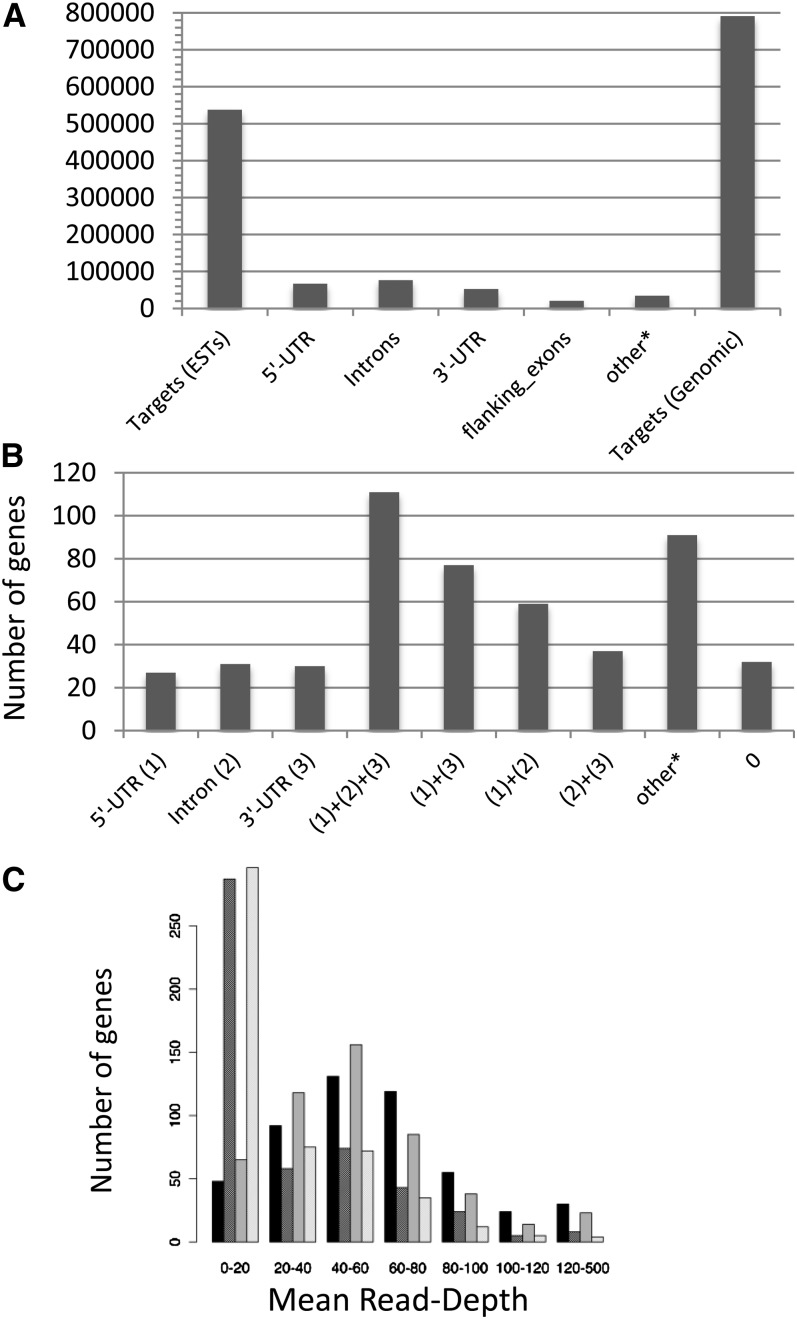
(A) Length of sequenced regions. Relative lengths of 5′ UTR, 3′ UTR, and intronic are shown in base pairs. The target (genomic) is the sum length of the reconstructed genomic sequence including the captured sequence flanking the direct targets. (B) Distribution of the number of genes with recovered genomic regions. ^*^Recovered untranslated genomic regions in unclosed introns or for genes displaying multiple gene models (C) Distribution of the mean read depth for Maxxa and TX-2094 of the captured reads mapped to the 500 targeted gene pairs. Bars, from darkest to lightest: mean read depth for Maxxa exons, Maxxa introns, TX2094 exons, and TX2094 introns.

When the *de novo* read assemblies were used, at least one nontargeted genomic region (flanking or intronic) was recovered for 463 of the targeted gene pairs. These flanking and intronic sequences facilitated reconstruction of genomic sequence from the *de novo* assembled captured reads (Figure 4). When this genomic reconstruction was used as a reference for read mapping, it increased the number of mapped bases compared with the ‘exonic reference’ mapping by the inclusion of previously trimmed bases (not additional sequencing reads). All possible classes of nontargeted, contiguous sequence, *i.e.*, 5′-UTR, intronic, flanking exonic, and 3′-UTR, were recovered for 131 gene pairs, whereas for the other 332 targets at least one of these regions was recovered (Figure 5). A total of 67,435 bp of 5′-UTR, 21,083 bp of flanking exon, 78,861 bp of intronic, and 52,722 bp of 3′-UTR was assembled, allowing the reconstruction of a genomic reference sequence of 791,241 bp for the targeted genes captured from the EST-derived probes (Figure 5). In all cases, the gene model inferred was validated by comparison to *de novo* gene model prediction using the genemark software (Table S3). The distribution of the mean sequence coverage for both translated and untranslated regions was similar between Maxxa and TX2094 after mapping the captured reads to this reconstructed genomic reference sequence (Figure 5C). The untranslated region coverage is >20× for 212 and 203 targeted genes for Maxxa and TX2094, respectively.

### SNP discovery and homeolog and allele assignment for single-copy genes

**Figure 6:**
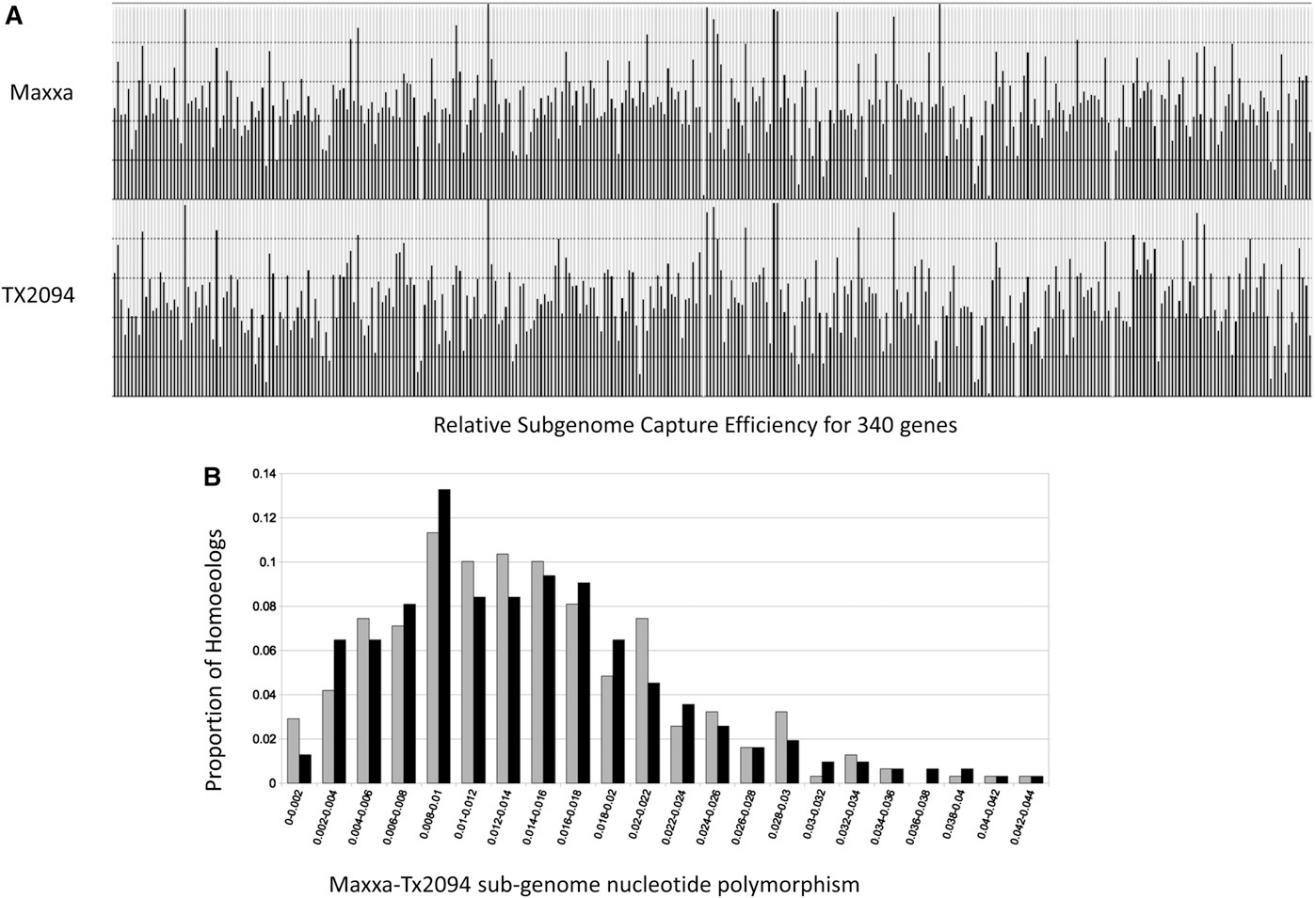
(A) Relative capture efficiencies of both homeologs (A-genome in gray; D-genome in black) in Maxxa (top) and TX2094 (bottom) for 342 single-copy genes from alignments to the ‘genomic’ reference (97% minimum overlap identity mapping); (B) Distribution of nucleotide polymorphism between Tx2094 and Maxxa (A_T_-genome in gray; D_T_-genome in black) for captured homeologs.

Many SNPs were detected in the alignments of mapped captured reads to the 411 translated regions and to the 411 reconstructed genomic reference sequences. A total of 1795 and 2166 were diagnostic of a homoeoSNP for the EST-based and the genomic reference enriched in flanking-exon sequences, respectively. These homoeoSNPs were used to assign reads from the allopolyploid *G. hirsutum* to genome-of-origin. This strategy enabled the detection of 347 A_T_ and D_T_ homeologs in Maxxa and 342 A_T_ and D_T_ homeologs for TX2094 (Table 4). This result allowed the calculation of the polymorphism rate between the A_T_- and D_T_-genomes within the two tested accessions (Figure 6B). Thus, the sequence capture strategy was highly effective in recovering homeologs with equal and high efficiency from both genomes (Figure 6A) and from both accessions.

**Table 4:** Number of targeted gene pairs for which both the A_T_ and D_T_ homeologs were detected (and within subgenomes, number of heterozygous loci), based on a *de novo* genomic reference sequence (Genomic) and the EST-based exonic reference (Exonic) sequence (97% of minimum overlap identity mapping)

	Accession	Genome	Exonic	Genomic
Whole dataset (500 targets)	TX2094	A_T_	404	411
		D_T_	400	409
	Maxxa	A_T_	413	415
		D_T_	410	416
Single copies (411 targets)	TX2094	A_T_ (He)	336 (10)	342 (21)
		D_T_ (He)	332 (13)	340 (25)
	Maxxa	A_T_ (He)	345 (0)	347 (1)
		D_T_ (He)	343 (5)	347 (17)

EST, expressed sequence tag.

Sequence capture was successful in detecting the two types of alleleSNPs. First, allele-SNPs between Maxxa-TX2094 (where each accession was homozygous) were detected (26 in TX2094 and 5 in Maxxa; Table S4). Second, alleleSNPs were detected within accessions where multiple bases were present within the sequences representing a single homeolog (62 in TX2094 and 21 in Maxxa; Table S4). This second type of alleleSNP indicated base positions within Maxxa and TX2094 that were heterozygous. As expected, a greater level of heterozygosity was detected in TX2094 than in Maxxa (Table 4; Fisher's exact tests: A_T_-genomes, *P* < 0.001). Interestingly, D_T_-genome loci were more heterozygous than their A_T_-genome homeologs in the cultivar Maxxa (Fisher's exact tests: *P* < 0.001). The number of reads that mapped to the reconstructed references (exonic/genomic) and the numbers of reads assigned to a given copy, and the mean coverage are presented in Table S4.

## Discussion

One of the challenges for use of the information embedded in complex plant genomes is the ability to detect and identify orthologous duplicated sequences, or homeologs. This is particularly problematic in polyploids of relatively recent origin, in that homeologous copies bear high sequence identity. Historically, methods for homeolog isolation and diagnosis have relied upon conventional approaches that entail PCR, cloning, and Sanger sequencing of selected clones. The labor-intensive nature of these methods places practical limitations on the number of homeologous pairs that can reasonably be included in many applications, often resulting in experiments with scales ranging from a few genes to tens of genes. Since the advent of next-generation sequencing methods, several new and methodologically powerful target enrichment strategies have been developed, such as RAD sequencing (Davey and Blaxter 2010), pooled amplicon sequencing (Griffin *et al.* 2011), and sequence capture (reviewed in Cronn *et al.* 2012; Grover *et al.* 2012; Mamanova *et al.* 2010; Mertes *et al.* 2011). These new methods provide a powerful means for catapulting many types of studies from the population genetics into population genomics arena. The increasing application of these approaches will likely transform diverse fields, including evolutionary and ecological studies, to which genomic or even epigenomics approaches were not, until recently, feasible (reviewed in Cronn *et al.* 2012 and Grover *et al.* 2012).

Although exome resequencing in primates has been extensively developed (Bainbridge *et al.* 2010; Cosart *et al.* 2011;George *et al.* 2011; Hodges *et al.* 2007, 2009; Ng *et al.* 2009; T1GP Consortium 2011), only a few studies have been reported to date in plants. In maize, sequence capture approaches by hybridization allowed the capture of 2.3 Mb and 43 genes between two maize lines (Fu *et al.* 2010). More recently, target enrichment has been used in soybean for exome resequencing of two accessions for SNPs discovery (Haun *et al.* 2011), and in allotetraploid wheat for exome resequencing of 3,497 genes (representing 3.5 Mb) for two accessions (Saintenac *et al.* 2011).

Here we report the ability to simultaneously capture hundreds of loci for multiplexed samples. This hybridization-based approach, which reduces genomic sample complexity, allowed the enrichment in targeted loci by more than 300-fold. This target enrichment results in an increased read-depth of the targeted loci, a critical consideration for validating the existence of *bona fide* SNPs, for confident detection of appropriate assignment of alleles for heterozygous loci, and for diagnosis of the genomic origin of homeologous genes. We tested the use of barcoded (MID-tagged) 454 libraries in different mixture proportions (50:50 and 75:25) and obtained sequenced reads from these libraries at the expected proportions, demonstrating the feasibility of using multiplexed sequence capture libraries for larger-scale experiments. We show that both homeologs from the two accessions studies were captured with equivalent efficiency, even though the hybridization probes were designed from EST assemblies from parental and various *G. hirsutum* cultigens (including the Acala Maxxa accession) EST libraries (Udall *et al.* 2006) and from a single consensus sequence of the targeted homeologous loci.

Although the purpose of the present study was largely methodological, we draw attention to one surprising and perhaps notable biological conclusion. Specifically, we note that heterozygosity levels are greater in the wild accession (TX2094) than in the cultigen (Maxxa), as expected given the many generations of inbreeding (generalized and actual) during cotton cultivar development (Table 4). In addition, though, the drop in heterozygosity in Maxxa is strikingly biased with respect to genome, such that for the A_T_ genome only a single heterozygous locus was detected. The difference in heterozygosity between the two genomes of Maxxa is dramatic, especially given the observation that A_T_ and D_T_ heterozygosity levels are equivalent in TX2094. Further studies are needed to explain this intriguing suggestion of differential selection accompanying domestication in the two genomes of modern Upland cotton.

The foregoing results have broad applicability, requiring only the information present in EST sequences from the species (or clade) of interest, although as yet there is no information that addresses the rate of drop-off of target locus recovery with increasing taxonomic or genetic divergence. One key factor in target design is that probes should avoid repeated sequences, so as not to waste sequencing space on redundant and often extraneous sequences. In many cases, even for nonmodel species, comparative genomics approaches using the phylogenetically closest available genome(s) should allow copy number estimations of each target. As shown here, when steps are taken to focus on low-copy sequences, the recovery rate for targeted genes can be very high.

One advantage of the methodology adopted here is that even though the probes were designed against expressed genes (and hence exons), captured regions can be bioinformatically extended into 5′ and 3′ flanking regions as well as introns. These noncoding regions typically are more rapidly evolving than exonic nucleotide sites and thus are of particular interest for polymorphism detection and diversity analyses. One can imagine a several-step sequence capture process to resequence coding and noncoding loci for species for which reference genomes are not available, with a first capture using targeting probes designed from ESTs, subsequent sequencing of captured libraries allowing the detection of untranslated sequences, and a second step using redesigned probes that both exclude the capture of undesirable repetitive sequences but which also include the previously recovered non-coding sequences. This second capture design would then be useful for larger-scale applications using an expanded set of samples. The net consequence of such an approach would be execution of massively parallel chromosome walking via sequence capture.

Because of these many advantages, targeted sequence capture approaches provide a powerful new tool for plant breeding, analyses of the scale and scope of genomic diversity, and a host of evolutionary and ecological genomic studies. By making diversity assessment accessible for genes throughout the genome, it becomes more feasible, for example, to detect the targets of natural and artificial selection. This technology, therefore, should find widespread application in analyses of crop domestication (reviewed in Doebley *et al.* 2006; Gross and Olsen 2010; Wright and Gaut 2005). With respect to cotton, in particular, domestication has been historically replicated, in parallel, for two diploid, A-genome species in the Old World (*G. herbaceum* and *G. arboreum*) and for two tetraploid cottons (AADD genomes) in the New World (*G. hirsutum* and *G. barbadense*). Cotton epidermal seed trichomes (commonly known as “fibers”) in domesticated cottons and their wild relatives differ by various morphological and structural characters, including density, length, cell growth synchronicity, shapes, and developmental shifts during the “fiber” elongation phase (Applequist *et al.* 2001). In polyploid cotton, domestication has been associated with selection for long, strong fibers, synchrony in fiber development, higher fiber cover, and more fiber initials on the day of anthesis (Butterworth *et al.* 2009). These transformations are concomitant with a massive repatterning of gene expression (Rapp *et al.* 2010) when comparing ‘wild’ (*e.g.*, TX2094) to improved (*e.g.*, Acala Maxxa) accessions. In the present study, we chose a subset of genes implicated as either affected by or involved in the domestication process. By studying a larger set of accessions spanning the wild to advanced continuum, it should be possible to use population genomic strategies to detect the genomic regions, and possibly the genes, that were unknowingly targeted by humans during the domestication process.

## Supplementary Material

Supporting Information
