# Supplementary material for: Targeted Capture of Homoeologous Coding and Noncoding Sequence in Polyploid Cotton
Source: G3 (Bethesda). 2012 Aug 1;2(8):921–30. doi: 10.1534/g3.112.003392 (PMC3411248; doi:10.1534/g3.112.003392)
Supplement: Supporting Information [file supp_2_8_921__index.html]

Supporting Information 

# Targeted Capture of Homoeologous Coding and Noncoding Sequence in Polyploid Cotton

## Supporting Information for Salmon *et al.*, 2012

**Files in this Data Supplement:**

- Table S1 - List of the 500 targeted gene pairs, EST sequence ids and functional annotation (.xls, 195 KB)
- Table S2 - Copy Number detection in the D5 genome (Gossypium raimondii) and intron detection (.xls, 119 KB)
- Table S3 - Gene model annotation from the de novo-built genomic reference using the genemark software (.xls, 216 KB)
- Table S4 - Exonic and de novo�built genomic mapping results: number of mapped reads per accession, SNP positions (.xls, 1.1 MB)
